# DeBi: Discovering Differentially Expressed Biclusters using a Frequent Itemset Approach

**DOI:** 10.1186/1748-7188-6-18

**Published:** 2011-06-23

**Authors:** Akdes Serin, Martin Vingron

**Affiliations:** 1 Max Planck Institute for Molecular Genetics, Ihnestrasse 63-73, 14195 Berlin, Germany

## Abstract

**Background:**

The analysis of massive high throughput data via clustering algorithms is very important for elucidating gene functions in biological systems. However, traditional clustering methods have several drawbacks. Biclustering overcomes these limitations by grouping genes and samples simultaneously. It discovers subsets of genes that are co-expressed in certain samples. Recent studies showed that biclustering has a great potential in detecting marker genes that are associated with certain tissues or diseases. Several biclustering algorithms have been proposed. However, it is still a challenge to find biclusters that are significant based on biological validation measures. Besides that, there is a need for a biclustering algorithm that is capable of analyzing very large datasets in reasonable time.

**Results:**

Here we present a fast biclustering algorithm called DeBi (Differentially Expressed BIclusters). The algorithm is based on a well known data mining approach called frequent itemset. It discovers maximum size homogeneous biclusters in which each gene is strongly associated with a subset of samples. We evaluate the performance of DeBi on a yeast dataset, on synthetic datasets and on human datasets.

**Conclusions:**

We demonstrate that the DeBi algorithm provides functionally more coherent gene sets compared to standard clustering or biclustering algorithms using biological validation measures such as Gene Ontology term and Transcription Factor Binding Site enrichment. We show that DeBi is a computationally efficient and powerful tool in analyzing large datasets. The method is also applicable on multiple gene expression datasets coming from different labs or platforms.

## Background

In recent years, various high throughput technologies such as cDNA microarrays, oligo-microarrays and sequence-based approaches (RNA-Seq) for transcriptome profiling have been developed. The most common approach for detecting functionally related gene sets from such high throughput data is clustering [1]. Traditional clustering methods like hierarchical clustering [2] and k-means [3], have several limitations. Firstly, they are based on the assumption that a cluster of genes behaves similarly in all samples. However, a cellular process may affect a subset of genes, only under certain conditions. Secondly, clustering assigns each gene or sample to a single cluster. However, some genes may not be active in any of the samples and some genes may participate in multiple processes.

Biclustering is a two-way clustering method for detecting local patterns in data. It finds subsets of genes that behave similarly in subsets of samples. Biclustering was initially introduced by Hartigan [4]. However, it was first applied by Cheng and Church [5] on gene expression data. Cheng and Church tried to identify submatrices of low mean residue score which indicates uniform fluctuation in expression profiles. Since the algorithm discovers one bicluster at a time, repeated application of the method on a modified matrix is needed for discovering multiple biclusters. This has the drawback that it results in highly overlapping gene sets. Ben-Dor et al. [6] detected a subset of genes whose expression levels induce the same linear ordering of the experiments. The drawback of this method is that it enforces a strict order of the samples. Bergmann et al. [7] identified biclusters which consist of the set of co-regulated genes and the conditions that induce their co-regulation. Murali and Kasif [8] found subsets of genes that are simultaneously similarly expressed across a subset of the samples. The algorithm uses prior knowledge about the sample phenotypes. Tanay et al. [9] defined biclustering as a problem of finding bicliques in a bipartite graph. Due to its high complexity, the number of rows the bicluster may have is restricted. Prelic et al. [10] defined the binary inclusion maximal biclustering (BIMAX) using a fast divide and conquer method. However, divide and conquer has the drawback of possibly missing good biclusters by early splits. Li et al. [11] developed an algorithm for discovering statistically significant biclusters from datasets containing tens of thousands of genes and thousands of conditions. Madeira and Oliveira have written a detailed review on different biclustering methods [12].

Here, we propose a novel, fast biclustering algorithm called DeBi that utilizes differential gene expression analysis. In DeBi, a bicluster has the following two main properties. Firstly, a bicluster is a maximum homogenous gene set where each gene in the bicluster should be highly or lowly expressed over all the bicluster samples. Secondly, each gene in the bicluster shows statistical difference in expression between the samples in the bicluster and the samples not in the bicluster. Differentially expressed biclusters lead to functionally more coherent gene sets compared to standard clustering or biclustering algorithms.

There are several advantages of the DeBi algorithm. Firstly, the algorithm is capable of discovering biclusters on very large datasets such as the human connectivity map data with 22283 genes and 6100 samples in reasonable time. Secondly, it is not required to define the number of biclusters a priori [5,7,10].

We evaluated the performance of DeBi on a yeast dataset [13], on synthetic datasets [10], on the connectivity map dataset which is a reference collection of gene expression profiles from human cells that have been treated with a variety of drugs [14], gene expression profiles of 2158 human tumor samples published by expO (Expression Project for Oncology), on diffuse large B-cell lymphoma (DLBCL) dataset [15] and on gene sets from the Molecular Signature Database (MSigDB) C2 category. We show that DeBi compares well with existing biclustering methods such as BIMAX, SAMBA, Cheng and Church's algorithm (CC), Order Preserving Submatrix Algorithm (OPSM), Iterative Signature Algorithm (ISA) and Qualitative Biclustering (QUBIC) [5-7,9,10].

## Results

We have evaluated our algorithm on six datasets (a) Prelic's benchmark synthetic datasets with implanted biclusters [10] (b) 300 different experimental perturbations of S. cerevisiae [13] (c) diffuse large B-cell lymphoma (DLBCL) dataset [15] (d) a reference collection of gene-expression profiles from human cells that have been treated with a variety of drugs [14] (e) gene expression profiles of 2158 human tumor samples published by expO (Expression Project for Oncology) (f) gene sets from the Molecular Signature Database (MSigDB) C2 category. The synthetic data is studied to show the performance of our algorithm in recovering implanted biclusters. Additionally, the effect of overlap between biclusters and noise on the performance of the algorithm can be studied using the synthetic data. The yeast and human gene expression datasets are studied to evaluate the biological relevance of the biclusters from several aspects. We used a fold-change of 2 for binarizing the datasets. The set of biclusters generated by all the algorithms are filtered such that the remaining ones have a maximum overlap of 0.5. (unless specified otherwise)

First, for each bicluster we calculated the statistically significantly enriched Gene Ontology (GO) terms using the hypergeometric test. We determined the proportion of GO term enriched biclusters at different levels of significance. Second, Transcription Factor Binding Sites (TFBS) enrichment is calculated by a hypergeometric test using transcription factor binding site data coming from various sources [16-18] at different levels of significance. The GO term and TFBS enrichment analyses are done using Genomica http://genie.weizmann.ac.il.

We have compared our algorithm with BIMAX, SAMBA, Cheng and Churchs algorithm (CC), Order Preserving Submatrix Algorithm (OPSM), Iterative Signature Algorithm (ISA) and Qualitative Biclustering (QUBIC) [5-7,9,10]. We used QUBIC software for QUBIC, BicAT software for OPSM, ISA, BIMAX and Expander software for SAMBA with default settings for each algorithm [10,19,20].

### Prelic's Synthetic Data

We applied our algorithm to a synthetic gene expression dataset. In the artificial datasets biclusters have been created on the basis of two scenarios (data available at http://www.tik.ee.ethz.ch/sop/bimax. In the first scenario, non-overlapping biclusters with increasing noise levels are generated. In the second scenario, biclusters with increasing overlap but without noise are produced. In both scenarios, biclusters with constant expression values and biclusters following an additive model where the expression values varying over the conditions are investigated.

In order to assess the performance of different biclustering algorithms, we used two measures from Prelic et al. [10] and Hochreiter et al. [21], respectively. The measure introduced by Prelic et al. calculates a similarity based on the Jaccard index between the computed biclusters and the implanted biclusters. Bicluster recovery score measures the accuracy of the predicted biclusters however it does not consider the number of biclusters in both sets. Hochreiter et al. introduced a consensus score by computing similarities between all pairs of biclusters and then assigning the biclusters of one set to biclusters of the other set. It penalizes different number of biclusters by dividing the sum of similarities by the numbers of biclusters in largest set. A more detailed description of the measures can be found in Additional File 1.

**Figure 1:**
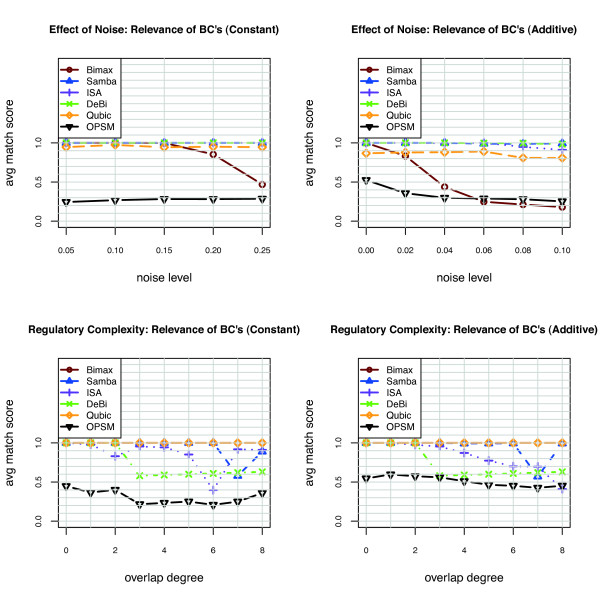
**Bicluster recovery accuracy score on synthetic data**. The synthetic data have been created based on two scenories (a) and (b) with increasing noise level, constant and additive model respectively. (c) and (d) with increasing degree of overlap, constant and additive model respectively.

**Figure 2:**
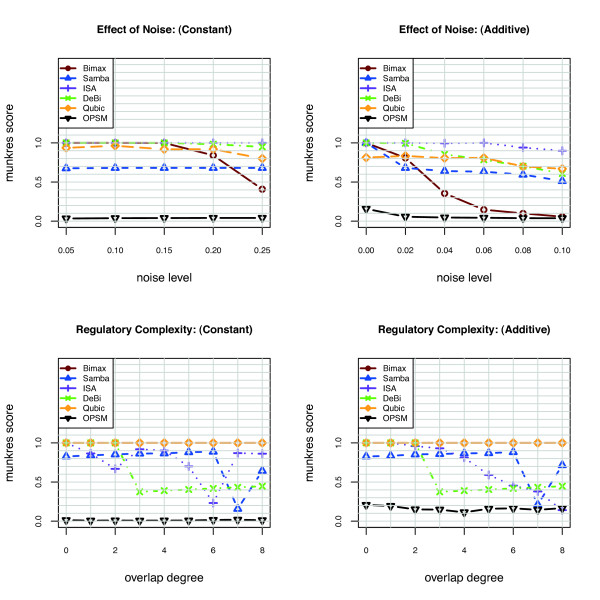
**Bicluster consensus score on synthetic data**. The synthetic data have been created based on two scenories (a) and (b) with increasing noise level, constant and additive model respectively. (c) and (d) with increasing degree of overlap, constant and additive model respectively.

In Figures 1 and 2 the performance of BIMAX, ISA, SAMBA, DeBi, OPSM and QUBIC algorithms on the synthetic data is summarized based on Prelic et al. recovery score and Hochreiter et al. consensus score. The set of biclusters generated by these algorithms are filtered such that the remaining ones have a maximum overlap of 0.25. In the Prelic et al. paper, after the filtering process the largest 10 biclusters are chosen. Since the bicluster number is not known a priori, we have considered all the filtered biclusters. We did not evaluate xMotif and CC algorithms since they have been shown to perform badly in all the scenarios, mostly below 50% of recovery accuracy [10]. The CC and xMotif algorithms produce large biclusters containing genes that are not expressed. ISA and QUBIC give high Prelic et al. recovery score and Hochreiter et al. consensus score in all scenarios. SAMBA has a lower Hochreiter et al. consensus score compared to its Prelic et al. recovery score. The reason is that, Hochreiter et al. consensus score takes into account both gene and condition dimensions and SAMBA is not very accurate in recovering the biclusters in condition dimension. In the absence of noise with an increasing overlap degree, BIMAX has a high performance based on Prelic et al. and Hochreiter et al. scores. However, BIMAX estimates a large number of biclusters upon increasing noise level. The comparision of the estimated number of biclusters given by the algorithms with the true number of biclusters under all the scenarios can be found in Figure S1 in Additional File 1. In the absence of overlap with increasing noise levels, DeBi is able to identify 99% of implanted biclusters both in additive and constant model. High degree of overlap decreases the performance of DeBi because it considers the overlapping part of the biclusters as a seperate bicluster. The DeBi biclustering results can be found in Additional file 2.

### Yeast Compendium

We further applied our algorithm to the compendium of gene expression profiles derived from 300 different experimental perturbations of *S. cerevisiae *[13]. We discovered 192 biclusters in the yeast dataset containing 2025 genes and 192 conditions. As a binarization level we used the fold change of 1.58 as recommended in the original paper [13].

**Figure 3:**
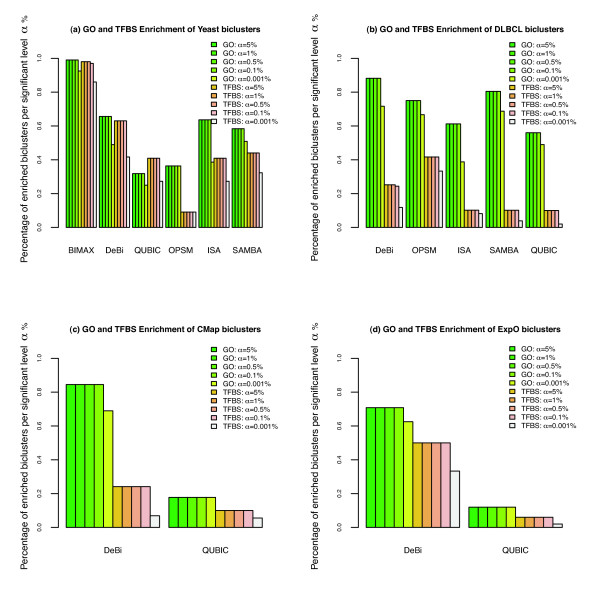
**Biological Significance of Yeast, DLBCL, CMap, ExpO Biclusters**. GO and TFBS enrichment of yeast, dlbcl, CMap and ExpO biclusters.

Figure 3 (a) illustrates the proportion of GO term and TFBS enriched biclusters for the six selected biclustering methods (ISA, OPSM, BIMAX, QUBIC, SAMBA and DeBi) at different levels of significance. DeBi performs the second best based on biological validation measures. BIMAX discovers a higher proportion of GO term and TFBS enriched biclusters. All the biclusters, the enrichment analysis can be found in Additional file 3.

In the analyzed yeast data, conditions are knocked-out genes. Since biclustering discovers subsets of genes and subsets of conditions we can also examine the biological significance of the clustered conditions. Similar to the previous analysis, we measured GO term enrichment of conditions in each discovered biclusters. DeBi is the second best in discovering high percentage of GO term enriched biclusters.

In the discovered biclusters, the enriched gene functions are related to the enriched sample functions. Bicluster 83, genes are enriched in the 'conjugation' GO term and conditions are enriched in 'regulation of biological quality'. Moreover, there is an enrichment of the TFBS of STE12, which is known to be involved in cell cycle. Bicluster 50, consists of genes and samples that are enriched in 'ribosome biogenesis and assembly' GO term. Bicluster 22, consists of genes and samples that are enriched in 'lipid metabolic process' GO term, and additionally genes are enriched with TFBS of HAP1. Bicluster 9, consists of down regulated genes and samples that are enriched in 'cell division' GO term, and additionally genes are enriched with TFBS of STE12.

### DLBCL Data

We also evaluated our DeBi algorithm on 'diffuse large B-cell lymphoma' (DLBCL) dataset. DLBCL dataset consists of 661 genes and 180 samples. We applied ISA, OPSM, QUBIC, SAMBA and DeBi algorithms.

Figure 3 (b) illustrates the proportion of GO term and TFBS enriched biclusters for the five biclustering methods at different levels of significance. DeBi discovers the highest proportion of GO term and TFBS enriched biclusters. The up regulated bicluster 16 and down regulated bicluster 4 contains the sample classes identified by [22]. Bicluster 16 is enriched with 'ribosome' and 'cell cycle' GO Term and Bicluster 4 is enriched with 'cell cycle' and 'death' GO Terms. The protein interaction networks of this two selected biclusters can be found in Figure S2 and S3 Additional File 1. Protein interaction networks are generated using STRING [23]. All the biclusters and the enrichment analysis can be found in Additional file 4.

### Human CMap Data

We also evaluated our DeBi algorithm on the Connectivity Map v0.2 (CMap) [14]. CMap is a reference collection of gene expression profiles from human cells that have been treated with a variety of drugs comprised of 6100 samples and 22283 genes. Figure 3 (c) summarizes the results of DeBi and QUBIC. The proportion of GO term and TFBS enriched biclusters are much more higher in DeBi compared to QUBIC.

**Figure 4:**
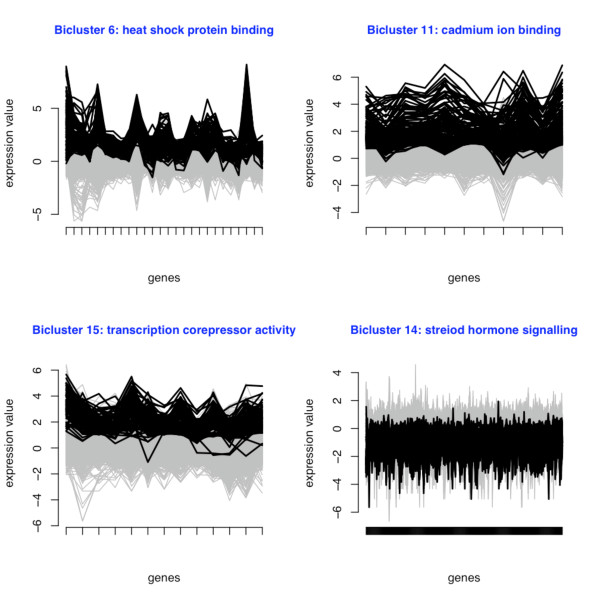
**Example CMap biclusters identified using DeBi Algorithm**. Parallel coordinate plots of some of the identified CMap biclusters using the DeBi algorithm. In parallel coordinate plots, the profile of the conditions that are included in a bicluster are shown as black, the other conditions as gray.

The biclusters discovered by DeBi can be used to find drugs with a common mechanism of action and identify new therapeutics. Moreover, we can observe the effect of drugs on different cell lines. Figure 4 shows parallel coordinate plots of some of the identified biclusters. In parallel coordinate plots, the profile of the conditions that are included in a bicluster are shown as black, the other conditions as gray. This aids to visualize the expression difference between the conditions in a bicluster compared to the rest of the conditions. The bicluster 6, contains up regulated 'heat shock protein binding' genes and 'heat shock protein inhibitors' such as geldanamycin, alvespimycin, tanespimycin, monorden. Heat shock proteins (Hsps) are overexpressed in a wide range of human cancers and are involved in tumor cell proliferation [24]. Additionally, genes in the bicluster are enriched with 'P53 binding site', which is known to target heat shock protein binding genes. Bicluster 11, contains up regulated genes enriched with 'cadmium ion binding' GO Term and calcium-binding protein inhibitors, calmidazolium. Bicluster 15, contains up regulated genes enriched with 'transcription corepressor activity' GO Term. Cell lines in this bicluster are all breast cancer. Bicluster 14, contains down regulated genes enriched with 'steroid hormone signalling' GO Term. Additionally, protein interaction networks of the selected biclusters are strikingly connected and they can be found in Figure S4, S5, S6 and S7 in Additional File 1. All the biclusters and the enrichment analysis can be found in Additional file 5.

### Human ExpO Data

We applied our DeBi algorithm and QUBIC on Expression Project for Oncology(expO) dataset http://www.intgen.org/. ExpO consists gene expression profiles of 2158 human tumor samples coming from diverse tissues with 40223 transcripts.

Figure 3 (d) shows that the proportion of GO term and TFBS enriched biclusters are much more higher in DeBi compared to QUBIC. It illustrates that DeBi performs better than QUBIC in ExpO data. 70% of the DeBi biclusters are enriched with GO Terms with a p-value smaller than 0.05. Moreover biclusters contain tumor samples mostly from similar tissue types. Figure S8 in Additional file 1 shows GO Term enrichment of some of the biclusters. Bicluster 13 contains thyroid tumor samples and genes enriched with 'protein-hormone receptor activity'. Bicluster 3 contains prostate tumor samples and genes enriched with 'tissue kallikrein activity'. Bicluster 22 contains mostly pancreas and colon samples and genes enriched with 'pancreatic elastase activity' GO Term. All the biclusters and the enrichment analysis can be found in Additional file 6.

### MSigDB Data

Finally, we applied our algorithm on the manually curated gene sets from the Molecular Signature Database (MSigDB) C2 category. The C2 category of MSigDB consists of 3272 gene sets in which 2392 gene sets are chemical and genetic pertubations and 880 gene sets are from various pathway databases. The gene sets naturally define a binary matrix where ones indicate the affected gene under certain pertubation/pathway. The binary matrix contains 18205 genes and 3272 samples. This analysis aids us to identify the pathways that are affected by chemical and genetic perturbations. It has not been possible to run QUBIC on this dataset while QUBIC requires a certain amount of overlap between genes.

**Figure 5:**
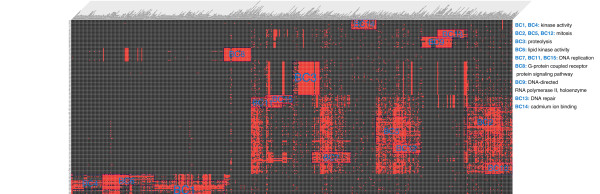
**MSigDB biclusters identified using DeBi Algorithm**.

Figure 5, illustrates all the biclusters using BiVoc algorithm [25]. BiVoc algorithm rearranges rows and conditions in order to represent the biclusters with the minimum space. The output matrix of BiVoc, may have repeated rows and/or columns from the original matrix. In Figure 5, the function of each bicluster is specified based on GO Term enrichment. Bicluster 3, contains the down-regulated gene set from Alzheimer patients and gene set from proteasome pathway. It is known that there is a significant decrease in proteasome activity in Alzheimer patients [26]. Bicluster 3 also contains the up-regulated gene set from pancreatic cancer patients. In previous studies, high activity of ubiquitin-proteasome pathway in pancreatic cancer cell line was detected [27]. Bicluster 8 contains up-regulated gene set from liver cancer patients and gene set from G-protein activation pathway. Dysfunction of G Protein-Coupled Receptor signaling pathways are involved in certain forms of cancer. All the biclusters and the enrichment analysis can be found in Additional file 7.

### Running Time

DeBi algorithm is capable of analyzing yeast data(size 6100 × 300) in 6 minutes, ExpO data (size 40223 × 2158) in 12 minutes, MSigDB data (size 18205 × 3272) in 11 minutes, DLBCL data (size 610 × 180) in 11 seconds, CMap data (size 22283 × 6100) in 3 hours 45 minutes. The QUBIC algorithm analyzes CMap data in 2 hours 55 minutes and ExpO data in 3 hours 54 minutes. The running time analysis was done on a 2.13 GHz Intel 2 Dual Core computer with 2GB memory.

## Methods

**Figure 6:**
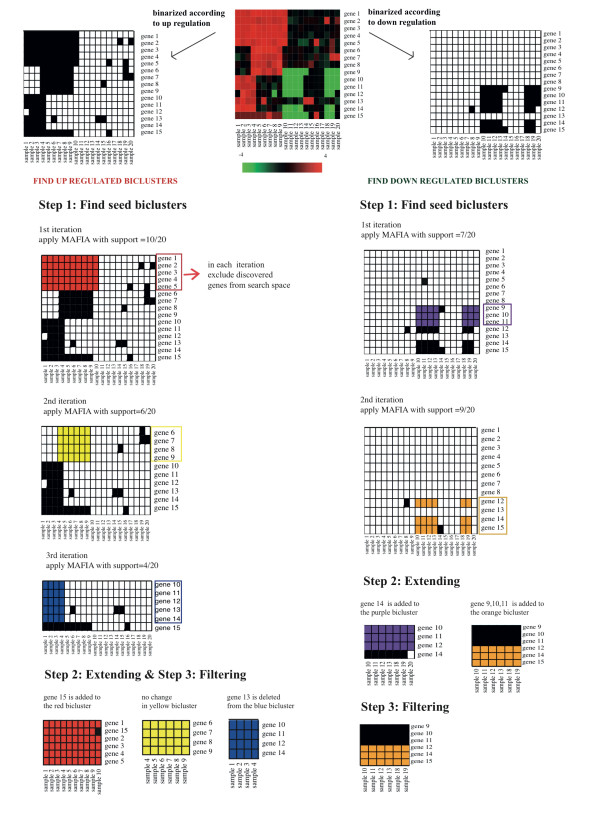
**Illustration of DeBi algorithm**. The algorithm is ran on two different binarized datasets. One is the binarized data based on up regulation and the other is the binarized data based on down regulation. In Step 1, seed biclusters identified within each support value going from high to low. For the binarized data based on up regulation, in the 1st iteration, red gene set with support value 10/20 is detected and excluded from the search space. Similarly, in the second and third iterations yellow and blue clusters with support values, respectively 6/20 and 4/20, are found. In Step 2, seed gene sets are improved based on genes' association strength. Gene 15 is added to the red bicluster because the p-value returned by the Fisher exact test is smaller than *α *and gene 13 is deleted because the p-value returned by the Fisher exact test is higher than *α*. None of the discovered biclusters have an overlap of the gene × sample area of more than 50%.

Given an expression matrix *E *with genes *G *={*g*_1_, *g*_2_, *g*_3_,..., *g_n_*} and samples *S *={*s*_1_, *s*_2_, *s*_3_,..., *s_m_*} a bicluster is defined as *b *= (*G'*, *S'*) where *G' *⊂ *G *is a subset of genes and *S' *⊂ *S *is a subset of samples. DeBi identifies functionally coherent biclusters *B *={*b*_1_, *b*_2_, *b*_3_,..., *b_l_*} in three steps. Below we describe each step in detail. An overview of the DeBi algorithm is shown in Figure 6. The DeBi algorithm is based on a well known data mining approach called Maximal Frequent Item Set [28]. We will refer to this as Maximal Frequent Gene Set, as given by our problem definition. The pseudocode of the algorithm is in Additional file 1.

### Preliminaries

The input gene expression data is binarized according to either up or down regulation. Let *E^u ^*and *E^d ^*denote the up and down regulation binary matrices, respectively. Then the entries  of *E^u ^*are defined as follows:(1)

and the entries of  of *E^d ^*are defined analogously with a c-fold down-regulation cut-off. The fold change cut-off *c *will typically be set to 2.

### Finding seed biclusters by Maximal Frequent Gene Set Algorithm

The DeBi algorithm, identifies the seed gene sets by iteratively applying the maximal frequent gene set algorithm. We first define the term *support*, which we will later use in the algorithm. The *support *of the gene *g_i_*, *i *= *1 *,..., *n*, is defined as follows:(2)

In other words, the *support *is the proportion of samples for which the gene-vector *e_i_*. is 1. This is further extended to sets of genes. Let  be the *v^th ^*gene-set. For a set of gene-vectors we define their *phenotype vector C_v _*as their element-wise logical AND:(3)

The *support *of the gene set is then defined as the fraction of samples for which the phenotype vector is 1.

A gene set  is (*c*_*1*_, *c*_*2*_) - *frequent *iff its support *supp * is larger than *c*_*1 *_and the cardinality  above *c*_*2*_. When *c*_*1 *_and *c*_*2 *_are not in focus, we will simply speak of a frequent gene set. A gene set is *maximally frequent *iff it is frequent and no superset of it is frequent.

The simplest method for detecting maximally frequent gene sets is a brute force approach in which each possible subset of *G *={*g*_1_, *g*_2_, *g*_3_,..., *g*_*n*_} is a candidate frequent set. To find the frequent sets we count the support of each candidate set. The MAFIA algorithm is an efficient implementation for finding maximally frequent sets with support above a given threshold [28]. The search strategy of MAFIA uses a depth-first traversal of the gene set lattice with effective pruning techniques. It avoids exhaustive enumeration of all candidate gene sets by a monotonicity principle. The monotonicity principle states that every subset of a frequent itemset is frequent. It prunes the candidates which have an infrequent subpattern using this property.

In the first step of the DeBi algorithm, MAFIA is iteratively applied to the binary matrix successively reducing the support threshold. Initially, MAFIA is applied to the full binary matrix *E*^*u *^(*E*^*d*^) with support value (*c*_*1 *_)_*0 *_equal to support value of the gene with the highest support. In iteration *k*, MAFIA is applied with support value threshold of . The identified maximally frequent sets are added to the set of seed gene sets *B *and the genes in *B *are deleted from the binary matrix *E^u ^*(*E*^*d*^). In each iteration MAFIA is applied to the modified matrix . The process is repeated until a user defined *minumum support *parameter is reached.

### Extending and filtering the biclusters

In the second step of DeBi, the identified seed gene sets  are extended using a local search. For each bicluster , *v *= 1,...,*l*, we have the binary phenotype vector *C*_*v*_= ∧(*e*_1_,...,*e*_*k*_) = (*C*_*v*1_,...,*C*_*vm*_). The entries of *C*_*v *_indicate the indices of the bicluster samples. If , *j *= 1,...,*m *, i.e. that the sample *s_j _*belongs to the bicluster b*_v_*. The gene *g_i_*, *i *= *1*,..., *n*, is an element of gene set  if *e*_*i*_. is associated with *C_v_*. We evaluate the association strength between the phenotype vector of a bicluster and another gene using Fisher's exact test on a 2 × 2 contingency table. The cells of the contingency table count how often the four possibilities of the phenotype vector containing a 1 or a 0 and the gene-vector containing a 1 or a 0 occur. The Fisher's exact test then tests for independence in the contingency table and thus among the two vectors.

A gene *g_i_*, *i *= *1*,..., *n *is added, to the gene set  if the pvalue  returned by the Fisher exact test is lower than the parameter *α*. It gets deleted from *b_v _*if the probability is higher than *α *and added to *b_v _*if the probability is smaller than *α*. For this procedure the association probability  with the bicluster needs to be calculated for each gene. However, we reduce the computational effort using the monotonicity property of the hypergeometric distribution. We precompute cut-off values on the contingency table entries that yield a p-value just higher than *α*. Let *σ_1, IN _*and *σ_1, OUT _*denote the number of 1's a gene-vector has in the bicluster samples and the number of 1's a gene-vector has outside the bicluster samples, respectively. We find the minimal *σ_1, IN _*and maximal *σ_1, OUT _*at this border. Then, we apply Fisher's exact test only to those genes which have *σ_1, IN _*>*minσ*_*1, IN *_and *σ*_*1*, *OUT *_<*maxσ*_*1*, *OUT*_.

In the last step we turn to the sometimes very complicated overlap structure among biclusters. The goal is to filter the set of biclusters such that the remaining ones are large and overlap only little. The size of a bicluster is defined as the number of genes times the number of samples in the bicluster, . Two biclusters overlap when they share common samples and genes. The size of the overlap is the product of the number of common samples and common genes. To filter out biclusters that are largely contained in a bigger bicluster, we start with the largest bicluster and compare it to the other biclusters. Those biclusters for which the overlap to the largest one exceeds L% (typically 50%) of the size of the smaller one are deleted. This is then repeated starting with the remaining second-largest bicluster and so on.

### Choosing the optimum alpha parameter

To formulate an optimality criterion for *α *one requires an inherent measure of the quality of a set of biclusters. To this end, for a bicluster *v*, we define its score *I_v _*as the negative sum of the log p-values of the included genes, where the individual *p_g _*is the p-value from the Fisher exact test:(4)

However, this bicluster score *I_v _*depends on the size (number of genes × number of conditions) of the bicluster and in order to make it comparable between biclusters one needs to correct for the size. We compute the expected bicluster score through a randomization procedure. A large number, say 500, random phenotype vectors having the same number of 1s as the bicluster has conditions is generated. For these random phenotype vectors a Fisher exact test p-value with respect to each gene in the bicluster is computed. One obtains a random *I_v _*score by adding log p-values over the genes of the bicluster. The mean of these random bicluster scores is the desired estimator. Finally, a normalized *NI_v _*score is definded by dividing *I_v _*by this estimated mean and the total biclustering score *CS *is defined as the sum of *NI*_*v *_normalized scores of all discovered biclusters . This score serves to distinguish between different choices of *α*. The program is run under *α *= {10^-2^, 10^-3^,..., 10^-100^} and we choose the *α *that maximizes CS.

## Discussion

We have proposed a novel fast biclustering algorithm especially for analyzing large datasets. Our algorithm aims to find biclusters where each gene in a bicluster should be highly or lowly expressed over all the bicluster samples compared to the rest of the samples. Unlike other algorithms, it is not required to define the number of biclusters a priori. We have compared our method with other biclustering algorithms using synthetic data and biological data. We have shown that the DeBi algorithm provides biologically significant biclusters using GO term and TFBS enrichment. We have also showed the computational efficiency of our algorithm. It is shown that it is a useful and powerful tool in analyzing large datasets.

In spite of efforts by many authors, comparing the performance of biclustering algorithms is still a challenge [29]. Smaller biclusters have a higher chance to yield a coherent GO annotation, while larger biclusters would, of course, be more interesting to observe. Our *α *threshold influences this behavior. The optimized α threshold is smaller for larger number of samples which limits the number of genes that get accepted into a bicluster.

The binarization of the input data in order to obtain a boolean matrix is another key decision in our approach. In this we go along with many other authors and we think that it helps in applying biclustering to gene expression data coming from different labs or platforms. The hope is that our method will further contribute to establishing biclustering as a general purpose tool for data analysis in functional genomics.

## Implementation

The DeBi code is written in c++ programming language for UNIX environment. The MAFIA algorithm c++ code is used for calculating the maximally frequent item sets. The DeBi algorithm is freely available at http://www.molgen.mpg.de/~serin/debi/main.html.

## Competing interests

The authors declare that they have no competing interests.

## Authors' contributions

AS developed and implemented the algorithm. AS drafted the versions of the manuscript. MV supervised the work and development of ideas. MV contributed with discussion of the draft versions and critical review. Both authors have read and approved the final manuscript.

## Supplementary Material

Additional file 1 **Description of selected biclustering algorithms, description of MAFIA algorithm, protein protein interaction networks**. Click here for file

Additional file 2 **DeBi results on synthetic data**. Click here for file

Additional file 3 **DeBi, BIMAX, ISA, OPSM, SAMBA and QUBIC biclustering results and GO term, TFBS enrichment analysis of the genes and conditions in biclusters on yeast data**. Click here for file

Additional file 4 **DeBi, ISA, OPSM, SAMBA, QUBIC biclustering results and GO term, TFBS enrichment analysis of the genes on DLBCL data**. Click here for file

Additional file 5 **DeBi and QUBIC biclustering results and GO term and TFBS enrichment analysis of the biclusters on CMap data**. Click here for file

Additional file 6 **DeBi and QUBIC biclustering results and GO term and TFBS enrichment analysis of the biclusters on ExpO data**. Click here for file

Additional file 7 **DeBi biclustering results and GO term and TFBS enrichment analysis of the biclusters on MSigDB data**. Click here for file
