# Supplementary material for: DeBi: Discovering Differentially Expressed Biclusters using a Frequent Itemset Approach
Source: Algorithms Mol Biol. 2011 Jun 23;6:18. doi: 10.1186/1748-7188-6-18 (PMC3152888; doi:10.1186/1748-7188-6-18)
Supplement: Additional file 1 — Description of selected biclustering algorithms, description of MAFIA algorithm, protein protein interaction networks. [file 1748-7188-6-18-S1.PDF]

Supplementary File for DeBi: Discovering Differentially Expressed Biclusters using a Frequent Itemset Approach

1 Description of the selected algorithms

Given an expression matrix E with genes G =$\{g_1, g_2, g_3, \ldots, g_n\}$ and samples S =$\{s_1, s_2, s_3, \ldots, s_m\}$ a bicluster is defined as $b = (G', S')$ where $G' \subset G$ is a subset of genes and $S' \subset S$ is a subset of samples. In this section some of the selected biclustering methods are described.

1.1 The Cheng and Church Algorithm (CC)

Model: The value of each entry e_{ij} in the gene expression matrix can be described using an additive model such as:

$$e_{ij} = \mu + \alpha_i + \beta_j \tag{1}$$

where μ is the background value, α_i is the row adjustment and β_j is the column adjustment.

Goal: The algorithm tries to find biclusters with a minumum *mean squared residue score*, H. *Mean squared residue score* is a measure of the coherence of the rows and columns in the bicluster.

Bicluster type: The algorithm discovers biclusters with coherent values.

Bicluster discovery: The algorithm discovers one bicluster at a time.

Approach: They use a greedy algorithm to find the optimal biclusters.

1.2 Statistical Algorithmic Method for Bicluster Analysis (SAMBA)

Model: The expression data is modeled as a bipartite graph *G=(U,V,E)*. In the graph, U is the set of conditions, V is the set of genes and $(u, v) \in E$ if the expression level of v changes significantly in u. The edges are assigned to weights according to a statistical model, so that heavy subgraphs corresponds to biclusters with high likelihood.

Goal: The goal of the algorithm is to find statistically significant heavy subgraphs.

Bicluster type: The algorithm discovers biclusters with coherent evolutions.

Bicluster discovery: The algorithm discovers biclusters simultaneously.

Approach: They use exhaustive enumeration to find the optimal biclusters.

1.3 Iterative Signature Algorithm (ISA)

Goal: The ISA algorithm identifies biclusters which consist of the set of co-regulated genes and the conditions that induce their co-regulation. It means that for each sample the average expression value of all the genes in bicluster should be surprisingly high/low and for each gene the average expression value of all the samples in bicluster should be surprisingly high/low.

Bicluster type: The algorithm discovers biclusters with coherent values.

Bicluster discovery: The algorithm discovers biclusters simultaneously.

Approach: They use a greedy algorithm to find the optimal biclusters.

1.4 Qualitative Biclustering Algorithm (QUBIC)

Model: The expression data is modeled as a graph *F=(V,E)*. In the graph, V is the set of genes and the edges $e = (v_i, v_j) \in E$ are weighted based on the similarity between the two genes, g_i and g_j.

Goal: Given the expression matrix E with genes $G = \{g_1, g_2, g_3, \ldots, g_n\}$ and samples $S = \{s_1, s_2, s_3, \ldots, s_m\}$ a bicluster is defined as $b = (G', S')$. The goal of the algorithm is to find all the biclusters such that $min(|G'|, |S'|)$ is maximal and the consistency level of (G', S') is higher than a pre-specified value c, $0 < c < 1$. The goal of the algorithm is to find maximum size consistent biclusters.

Bicluster type: The algorithm discovers biclusters with coherent evolutions.

Bicluster structure: The algorithm discovers biclusters simultaneously.

Approach: They use exhaustive enumeration to find the optimal biclusters.

1.5 Order Preserving Sub-matrices Algorithm (OPSM)

Model: A bicluster is an ordering of the subset of samples such that the expression values of all genes in bicluster are sorted in ascending order.

Goal: The goal of the algorithm is to find order preserving sub-matrices of maximum statistical significance.

Bicluster type: The algorithm discovers biclusters with coherent evolutions.

Bicluster discovery: The algorithm discovers one bicluster at a time.

Approach: They use a greedy algorithm to find the optimal biclusters.

1.6 Binary Inclusion Maximal Algorithm (BIMAX)

Goal: The goal of the algorithm is to identify all maximal biclusters where none of the biclusters are not completely contained in any other bicluster.

Bicluster type: The algorithm discovers biclusters with constant values.

Bicluster structure: The algorithm discovers biclusters simultaneously.

Approach: They find the optimal biclusters using divide and conquer approach.

2 Validating biclustering results using synthetic data

In order to assess the performance of different biclustering algorithms, we used two measures from Prelic and Hochreiter, respectively. The measure introduced by Prelic et al. calculates the similarity between the computed biclusters and the implanted biclusters. Prelic score is defined as:

$$S_G(M, M_{opt}) = \frac{1}{|M|} \sum_{G \in M} \max_{G_{opt} \in M_{opt}} \frac{|G_{opt} \cap G|}{|G_{opt} \cup G|} \tag{2}$$

where M_{opt} is the set of true biclusters, M is the set of computed biclusters, G is the gene sets within the biclusters M and G_{opt} is the gene sets within the biclusters M_{opt}. The score $S_G(M, M_{opt})$ measures the relevance of the predicted biclusters in gene dimension. The maximum value for the score is 1. The drawback of this measure is that different number of biclusters of M_{opt} and M is not penalized.

Hochreiter et al. calculates the consensus score using the following steps. Firstly, given the two sets of biclusters M_{opt} and M, the similarities between all possible pairs of biclusters are computed. Secondly, each bicluster in M_{opt} is assigned to a bicluster in M using Munkres algorithm. Munkres algorithm

yields a matching between M_{opt} and M where no two pairings share the same bicluster. Finally, different number of biclusters are penalized by dividing the sum of similarities by the numbers of biclusters in largest set.

In the Prelic synthetic data we know apriori that 10 biclusters are implanted. Figure S1 compares the estimated number of biclusters with the true number of biclusters.

3 Different α parameters of DeBi in relation to estimated number of biclusters

Table S1, S2, S3 and S4 shows the estimated number of biclusters in relation to different α parameters of DeBi for the Prelic synthetic data. The DeBi algorithm selects the α that gives the maximum clustering score. The chosen α values are shown in red. In the Prelic synthetic data we know apriori that 10 biclusters are implanted. As it can be seen from the Table S1, S2, S3 and S4, DeBi always chooses the α value that gives the closest optimal bicluster number.

	Noise 0		Noise 0.05		Noise 0.1		Noise 0.15		Noise 0.2		Noise 0.25	
α	BicNum	BicScore	BicNum	BicScore	BicNum	BicScore	BicNum	BicScore	BicNum	BicScore	BicNum	BicScore
10^{-2}	10	230.58	10	231.14	10	232.6	10	226.63	10	214.25	10	207.14
10^{-3}	10	231.42	10	229.99	10	232.39	10	230.04	10	217.98	10	205.06
10^{-4}	10	231.42	10	230.4	10	232.39	10	229.41	8	175.58	3	65.61
10^{-5}	10	231.19	10	230.4	10	234.16	9	207.26	7	150.79	0	0
10^{-6}	10	232.72	10	231.67	10	233.53	3	72.7	0	0	0	0

Table 1: Constant Model: Increasing Noise Level

	Noise 0		Noise 0.02		Noise 0.04		Noise 0.06		Noise 0.0.08		Noise 0.1	
α	BicNum	BicScore	BicNum	BicScore	BicNum	BicScore	BicNum	BicScore	BicNum	BicScore	BicNum	BicScore
10^{-2}	10	233.31	10	204.98	9	175.57	8	98.61	8	105.63	8	115
10^{-3}	10	233.31	10	204.98	8	162.58	2	26.88	2	25.99	0	0
10^{-4}	10	234.77	9	185.17	7	140.36	0	0	0	0	0	0
10^{-5}	10	229.59	3	66.85	3	62.93	0	0	0	0	0	0
10^{-6}	10	226.15	0	0	0	0	0	0	0	0	0	0

Table 2: Additive Model: Increasing Noise Level

	Overlap 0		Overlap 1		Overlap 2		Overlap 3		Overlap 4		Overlap 5		Overlap 6		Overlap 7		Overlap 8	
α	BicNum	BicScore	BicNum	BicScore	BicNum	BicScore	BicNum	BicScore	BicNum	BicScore	BicNum	BicScore	BicNum	BicScore	BicNum	BicScore	BicNum	BicScore
10^{-2}	10	417.41	10	402.1	10	400.55	5	153.65	5	159.89	5	166.77	5	173.37	5	182.75	5	188.66
10^{-3}	10	416.05	10	409.1	10	399.33	5	150.51	5	158.64	5	166.77	5	170.98	5	182.36	5	187.12
10^{-4}	10	416.68	10	407.88	10	395.62	5	149.33	5	158.92	5	168.55	5	169.06	5	180.24	5	186.36
10^{-5}	10	416.68	10	410.27	10	400.69	9	344.18	9	389.43	5	168.89	5	176.95	5	181.32	5	189.31
10^{-6}	10	415.2	10	410.39	10	396.73	9	349.76	9	400.75	9	456.08	9	514.05	5	181.69	5	188.89
10^{-7}	10	417.92	10	406.6	10	402	9	347.3	9	403.07	9	460.18	9	504.32	9	573.06	9	664.37
10^{-8}	10	414.06	10	406.4	10	397.16	9	351.36	9	399.24	9	459.81	9	517.11	9	584.18	9	654.96
10^{-9}	10	419.13	2	96.31	10	395.34	9	346.55	9	394.87	9	434.66	9	519.04	9	578.47	9	648.85
10^{-10}	10	416.31	2	99.44	2	104.64	2	118.98	9	396	9	444.39	9	519.19	9	579.49	9	635.59
10^{-11}	10	416.75	2	101.29	2	104.82	2	125.96	2	134.8	9	451.22	9	503.68	9	589.02	9	654.5
10^{-12}	10	416.75	2	99.44	2	108.48	2	128.16	2	145.21	2	138.7	2	154.06	9	556.94	9	666.7
10^{-13}	10	411.93	2	101.29	2	104.82	2	125.96	2	145.21	2	150.54	2	158.9	2	162.72	9	653
10^{-14}	0	0	2	99.44	2	108.48	2	133.68	2	145.21	2	150.54	2	158.9	2	162.72	2	168.97
10^{-15}	0	0	0	0	2	104.82	2	125.96	2	145.21	2	150.54	2	158.9	2	151.28	2	168.97
10^{-16}	0	0	0	0	0	0	2	128.16	2	127.87	2	150.54	2	158.9	2	141.82	2	168.97
10^{-17}	0	0	0	0	0	0	0	0	0	0	2	133.53	2	135.96	2	141.82	2	168.97
10^{-18}	0	0	0	0	0	0	0	0	0	0	0	0	2	123.54	2	141.82	2	177.05

Table 3: Constant Model: Increasing Degree of Overlap

	Overlap 0		Overlap 1		Overlap 2		Overlap 3		Overlap 4		Overlap 5		Overlap 6		Overlap 7		Overlap 8		Overlap 9		Overlap 10	
α	BicNum	BicScore	BicNum	BicScore	BicNum	BicScore	BicNum	BicScore	BicNum	BicScore	BicNum	BicScore	BicNum	BicScore	BicNum	BicScore	BicNum	BicScore	BicNum	BicScore	BicNum	BicScore
10^{-2}	10	425.88	10	407.26	10	396.34	5	150.27	5	161.92	5	169.99	5	175.95	5	182.13	5	184.71	5	196.72	5	198.03
10^{-3}	10	416.22	10	403.52	10	391.91	5	149.53	5	158.15	5	165.47	5	174.87	5	180.4	5	186.92	5	192.68	5	202.47
10^{-4}	10	418.86	10	403.59	10	390.41	5	151.8	5	161.47	5	168.58	5	171.11	5	181.29	5	187	5	190.36	5	201.4
10^{-5}	10	418.25	10	405.02	10	392.51	9	348.19	9	395.47	5	172.48	5	173.55	5	183.8	5	184.44	5	198.01	5	194.14
10^{-6}	10	418.25	10	402.48	10	400.11	9	345.46	9	393.02	9	462.99	9	502.03	5	184.58	5	189.09	5	193.78	5	195.36
10^{-7}	10	413.75	10	404.12	10	404.71	9	354.69	9	398.93	9	450.98	9	532.33	9	580.92	9	658.75	9	754.98	5	199.09
10^{-8}	10	417.64	10	412.61	10	394.56	9	351.61	9	400.25	9	449.03	9	520.83	9	574.4	9	655.34	9	714.72	9	839.42
10^{-9}	10	421.23	2	100.87	10	386.61	9	350.08	9	391.78	9	454.61	9	514.38	9	599.46	9	635.26	9	700.72	9	825.94
10^{-10}	10	415.25	2	98.97	2	104.65	2	114.76	9	389.42	9	440.44	9	515.31	9	576.34	9	673.44	9	734.52	9	814.97
10^{-11}	10	418.26	2	100.87	2	101.17	2	121.2	2	127.65	9	453.02	9	511.19	9	582.41	9	656.49	9	725.81	9	848.04
10^{-12}	10	418.06	2	98.97	2	104.65	2	123.91	2	136.22	2	133.12	2	158.59	9	588.82	9	683.09	9	719.32	9	821.84
10^{-13}	10	413.2	2	100.87	2	101.17	2	121.2	2	136.22	2	140.09	2	165.46	2	151.92	9	654.04	9	735.53	9	844.34
10^{-14}	0	0	2	98.97	2	111.5	2	129.31	2	136.22	2	140.09	2	152.2	2	157.03	2	181.03	2	221.22	9	823.81
10^{-15}	0	0	0	0	2	105.56	2	121.2	2	136.22	2	140.09	2	152.2	2	153.64	2	181.03	2	221.22	11	1148.31
10^{-16}	0	0	0	0	0	0	2	123.91	2	120.65	2	140.09	2	152.2	2	153.64	2	181.03	2	221.22	11	1172.6
10^{-17}	0	0	0	0	0	0	0	0	0	0	2	124.53	2	136.99	2	153.64	2	181.03	2	221.22	11	1168.32
10^{-18}	0	0	0	0	0	0	0	0	0	0	0	0	2	125.42	2	153.64	2	180.67	2	221.22	11	1165.97

Table 4: Additive Model: Incerasing Degree of Overlap

4 Maximal Frequent Itemset Algorithm (MAFIA)

Identifying maximally frequent itemsets is a well known data mining problem. A typical application is on the customer market basket behavior analysis. In this example, the transaction database contains the collections of items bought by customers. The support of an itemset is the proportion of customers who bought the itemset. The goal is to find the maximal length itemsets that consumers buy frequently i.e. above a support value threshold. In our problem formulation, samples are the customers and items are the genes.

The simplest method for detecting maximally frequent itemsets is brute force approach in which each set in the transaction database is a candidate frequent set. To find the frequent sets we count support of each candidate set by scanning the transaction database. Since running time for the brute force approach is exponential, there is a need for more efficient implementations where we can reduce the number of candidates, number of transactions and number of comparisons.

MAFIA algorithm is an efficient implementation for finding maximal frequent itemsets, especially when the sets in the database are very long. The search strategy of MAFIA uses a depth-first traversal of the gene set lattice with effective pruning mechanisms. It avoids exhaustive enumeration of all candidate gene sets by monotonicity principle. In monotonicity principle, it is stated that every subset of a frequent itemset is frequent. It prunes the candidates which

have an infrequent sub pattern using this property.

5 DeBi Algorithm pseudocode

Given an expression matrix E with genes $G = \{g_1, g_2, g_3, \ldots, g_n\}$ and samples $S = \{s_1, s_2, s_3, \ldots, s_m\}$ a bicluster is defined as $b = (G', S')$ where $G' \subset G$ is a subset of genes and $S' \subset S$ is a subset of samples. DeBi identifies functionally coherent biclusters $B = \{b_1, b_2, b_3, \ldots, b_l\}$ in three steps. The input gene expression data E is binarized according to either up or down regulation. Let E^u and E^d denote the up and down regulation binary matrices, respectively. The DeBi takes E^u or E^d as an input.

Algorithm 5.1: DEBI($E^u(E^d), minc_1, c_2$)

comment: $minc_1$, minumum support value

comment: c_2, minumum num of genes

global $minc_1, c_2, overlap$

procedure FINDSEEDS(E^u)

$B \leftarrow \emptyset$

$E' \leftarrow E^u$

comment: c_1 set to support value of the gene with the highest support

$c_1 \leftarrow \max_i \frac{1}{m} \sum_{j=1}^{m} e_{ij}$

while $minc_1 \leq c_1$

do

- $B' \leftarrow \text{MAFIA}(E', c_1, c_2)$
- $I \leftarrow \emptyset$
- **for each** $b \in B'$
  - **do**
    - **comment:** get the genes in bicluster b
    - $I \leftarrow I \cup (G' \in b)$
- **comment:** remove the discovered genes from the expression matrix E
- $E' \leftarrow E' \backslash I$
- $B \leftarrow B \cup B'$
- $c_1 \leftarrow c_1 - 1$

return (B)

procedure EXTEND(E^u, B, α)

comment: *score* is the vector of bicluster scores

comment: for all the seed biclusters, $B = \{b_1, b_2, b_3, \ldots, b_l\}$

for $i \leftarrow 1$ **to** l

do
- **comment:** get the phenotype vector of bicluster i containing k genes
- $C_i \leftarrow \wedge(e_{1.}, \ldots, e_{k.})$
- **comment:** for all the genes in E^u
- **for** $j \leftarrow 1$ **to** n
- **do**
  - $pval \leftarrow$ FISHEREXACTTEST($C_i, e_{j.}$)
  - **if** $pval \leq \alpha$
  - **then**
    - **comment:** bicluster $b_i = (G'_i, S'_i)$
    - $G'_i \leftarrow G'_i \cup g_j$
    - $score[i] \leftarrow score[i] - \log(pval)$

return $(B, score)$

procedure BicScore($B, num, score$)

$CS \leftarrow 0$

for $i \leftarrow 1$ **to** l
- **comment:** get the phenotype vector of bicluster i with k genes
- $C_i \leftarrow \wedge(e_{1.}, \ldots, e_{k.})$
- **for** $j \leftarrow 1$ **to** num
  - $C'_i \leftarrow$ permute(C_i)
  - **comment:** bicluster $b_i = (G'_i, S'_i)$
  - **for each** $g \in G'_i$
    - **do**
      - $pval \leftarrow$ FisherExactTest($C'_i, e_{g.}$)
      - $sum[j] \leftarrow sum[j] - \log(pval)$
- $NS \leftarrow score[i]/mean(sum)$
- $CS \leftarrow CS + NS$

return (CS)

main

comment: *BicScore* is the vector of normalized biclustering scores for different α values

comment: *num* is the number of permutations

$B_1 \leftarrow \textsc{FindSeeds}(E^u(E^d))$

$maxScore \leftarrow 0$

for $i \leftarrow 2$ **to** 100

$$\textbf{do} \begin{cases} \alpha \leftarrow pow(10, -i) \\ (B_2, score) \leftarrow \textsc{Extend}(E^u(E^d), B_1, \alpha) \\ B_3 \leftarrow \textsc{Filter}(B_2, overlap) \\ BicScore[i] \leftarrow \textsc{BicScore}(B_3, score, num) \\ \textbf{if } maxScore \leq BicScore[i] \\ \quad \textbf{then} \begin{cases} maxScore \leftarrow BicScore[i] \\ maxAlpha \leftarrow \alpha \end{cases} \end{cases}$$

$B_2 \leftarrow \textsc{Extend}(B_1, maxAlpha)$

$B_3 \leftarrow \textsc{Filter}(B_2, overlap)$

output (B_3)

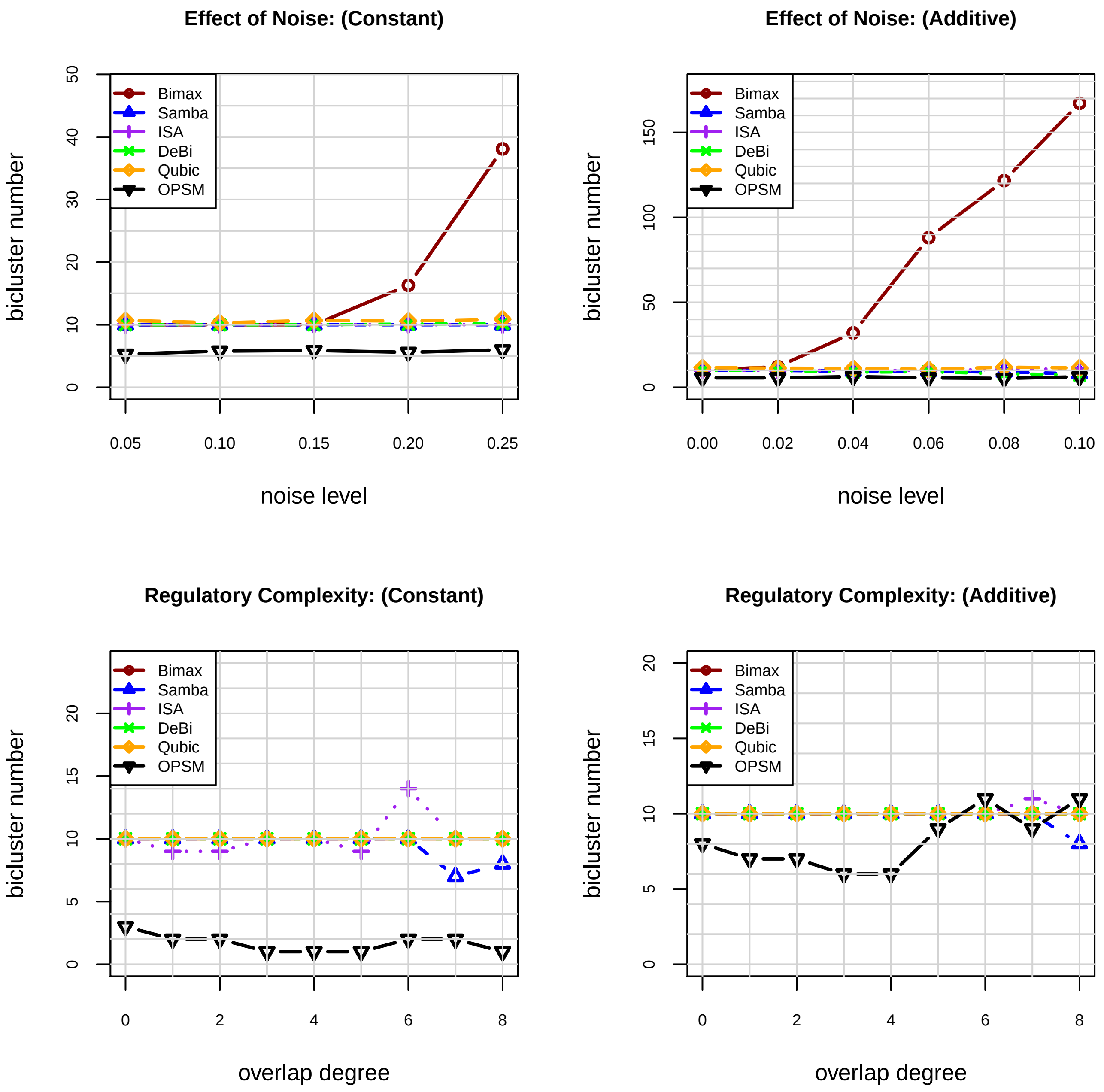

Figure 1: Comparision of the estimated number of biclusters with the true number of biclusters

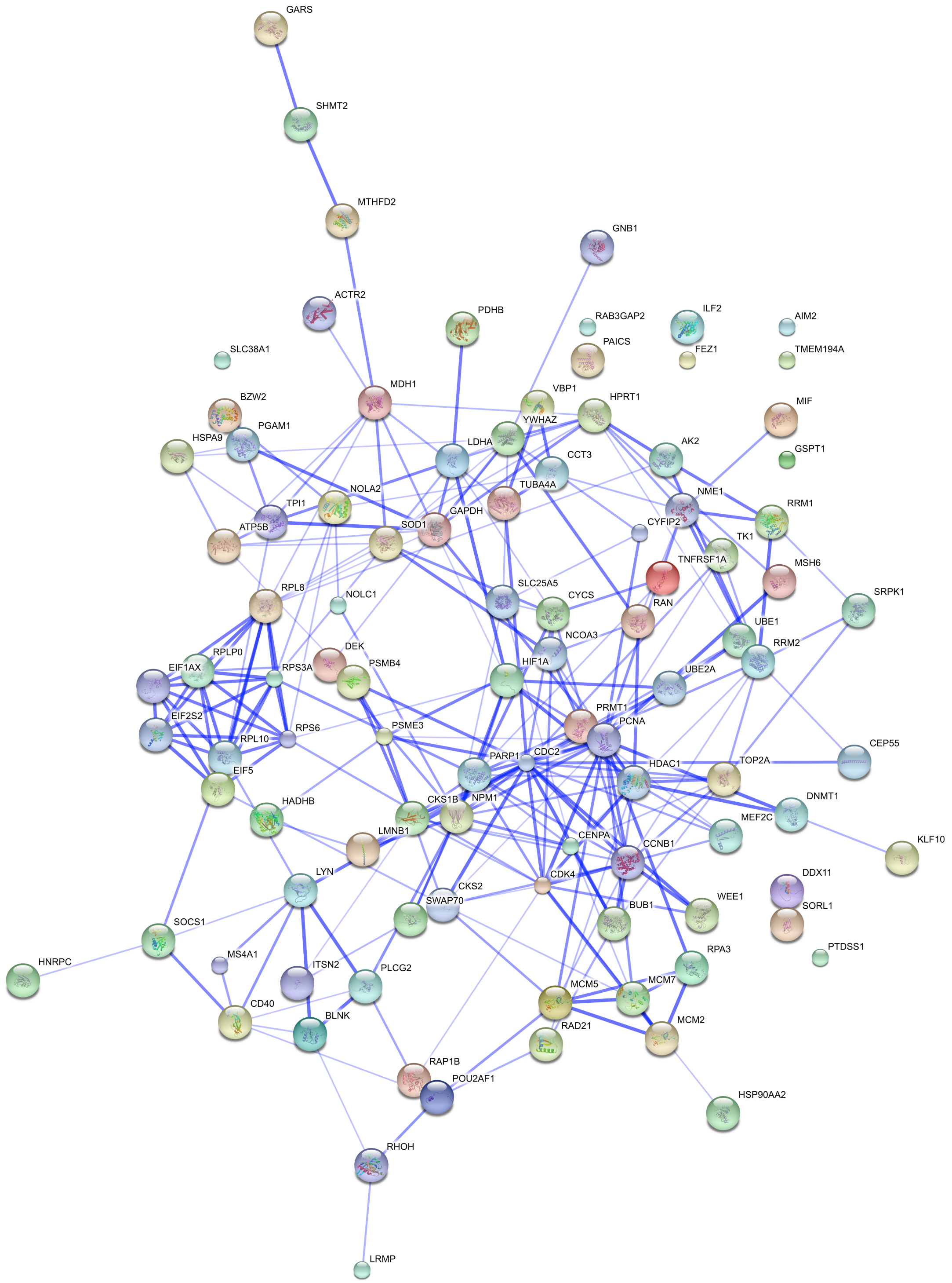

Figure 2: Bicluster 4-DLBLC Data

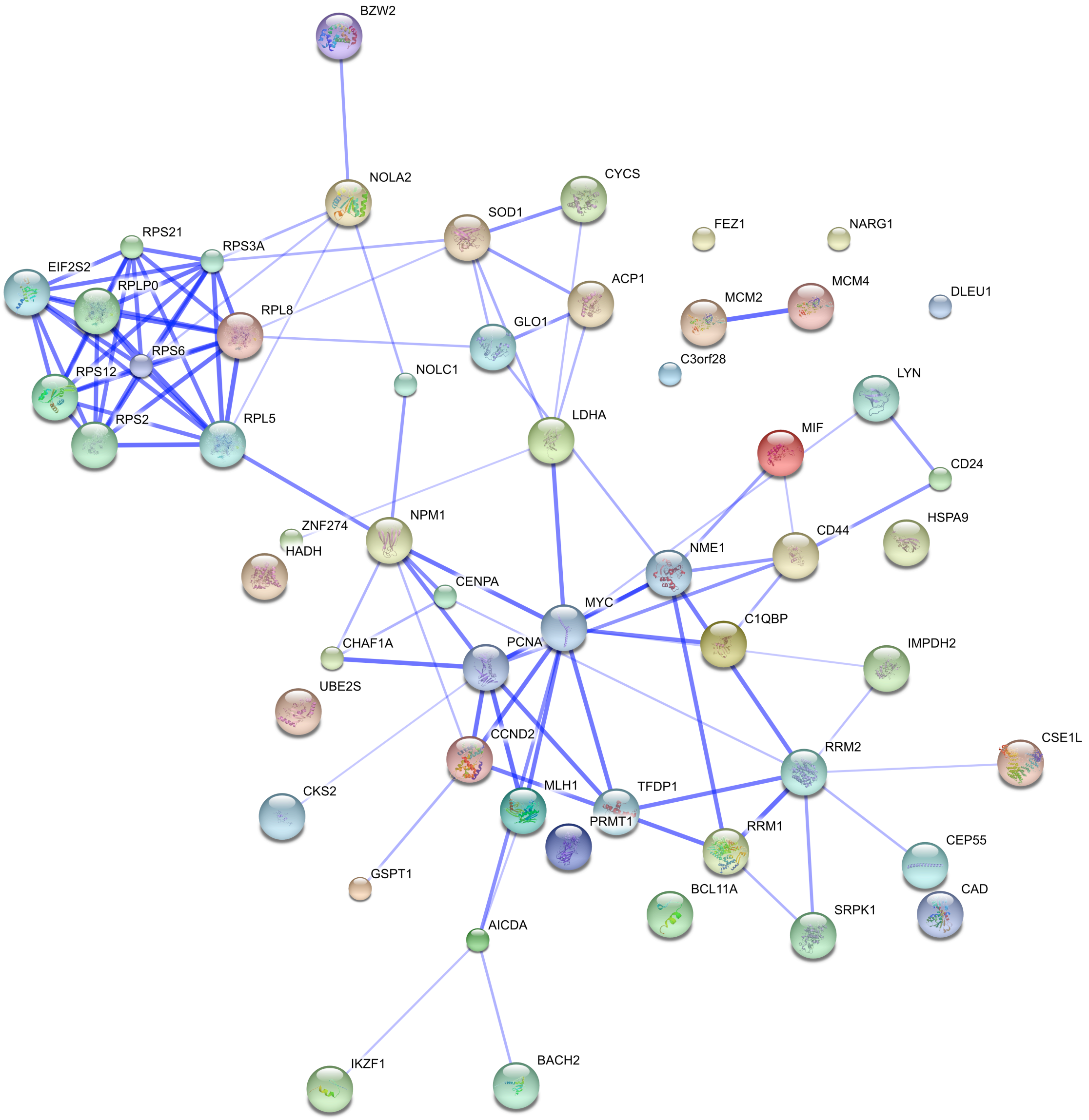

Figure 3: Bicluster 16-DLBLC Data

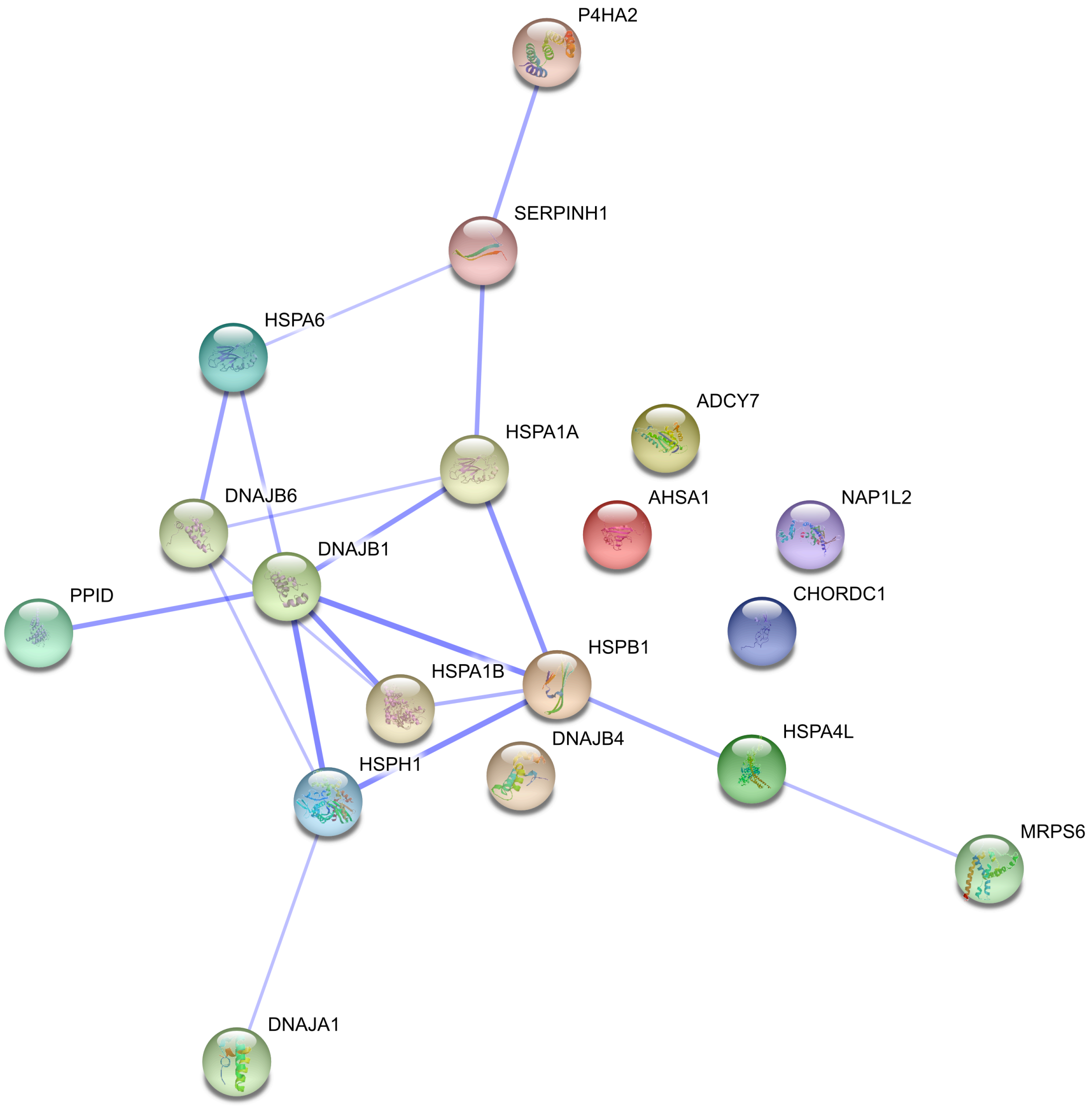

Figure 4: Bicluster 6-CMap Data

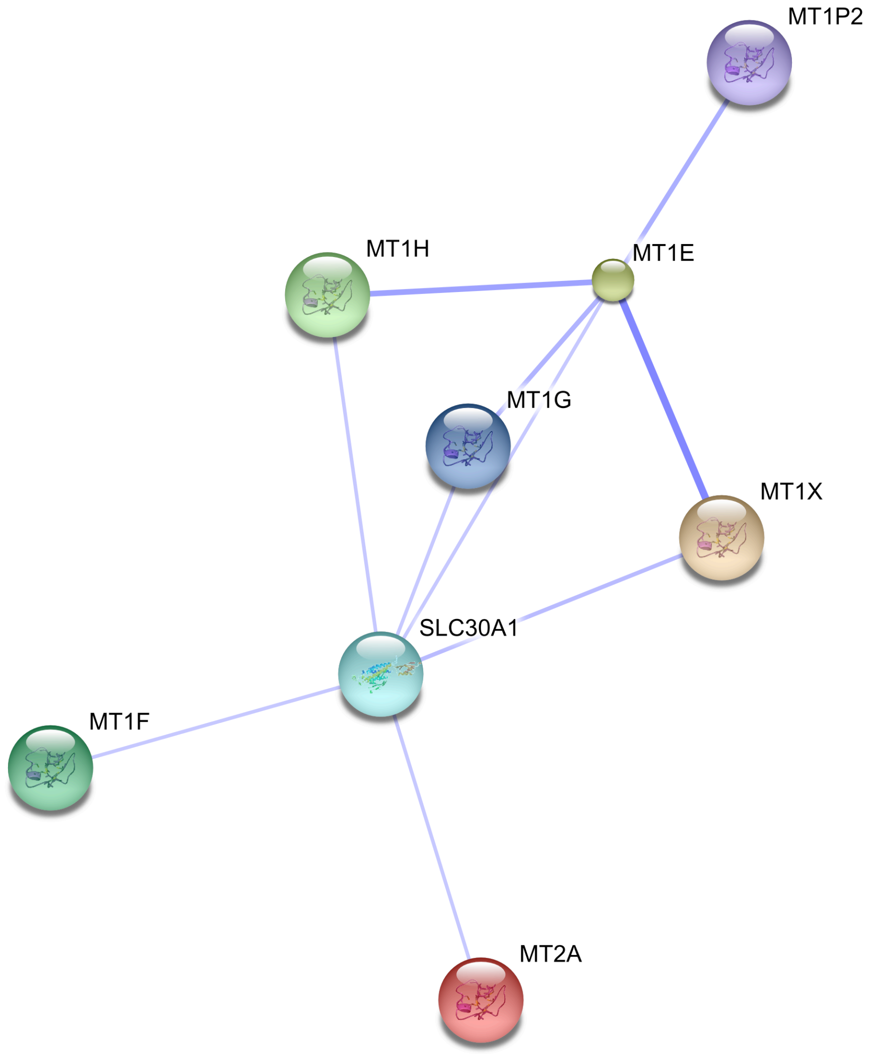

Figure 5: Bicluster 11-CMap Data

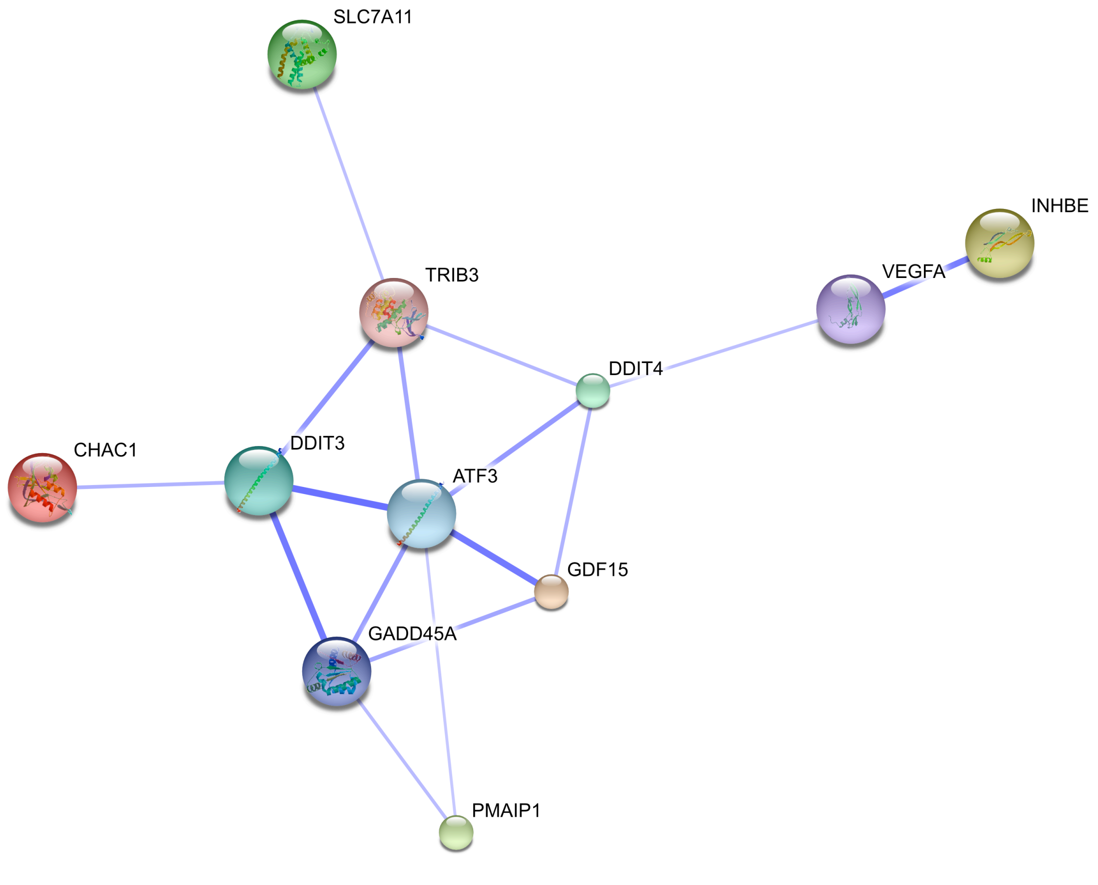

Figure 6: Bicluster 15-CMap Data

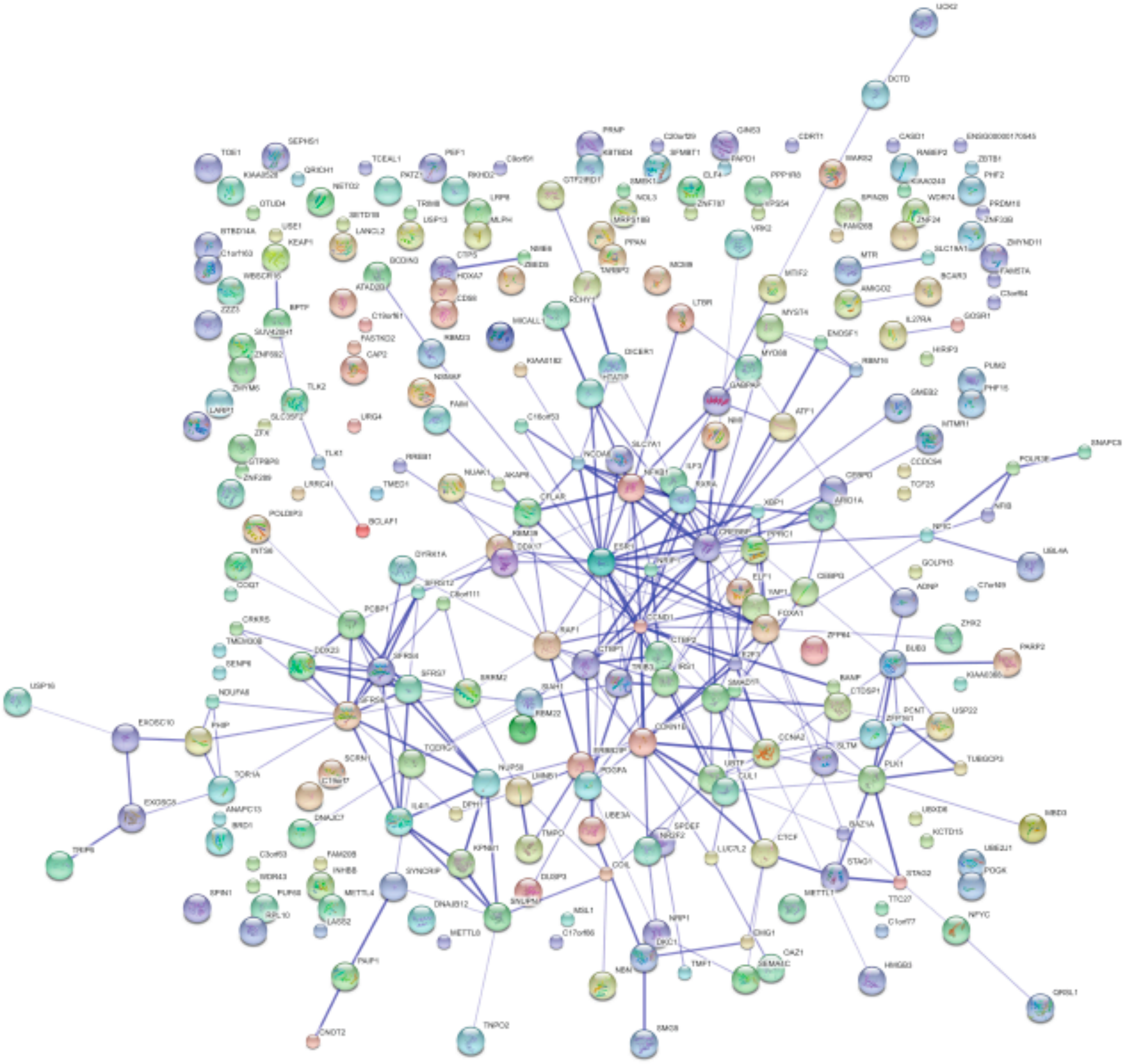

Figure 7: Bicluster 14-CMap Data

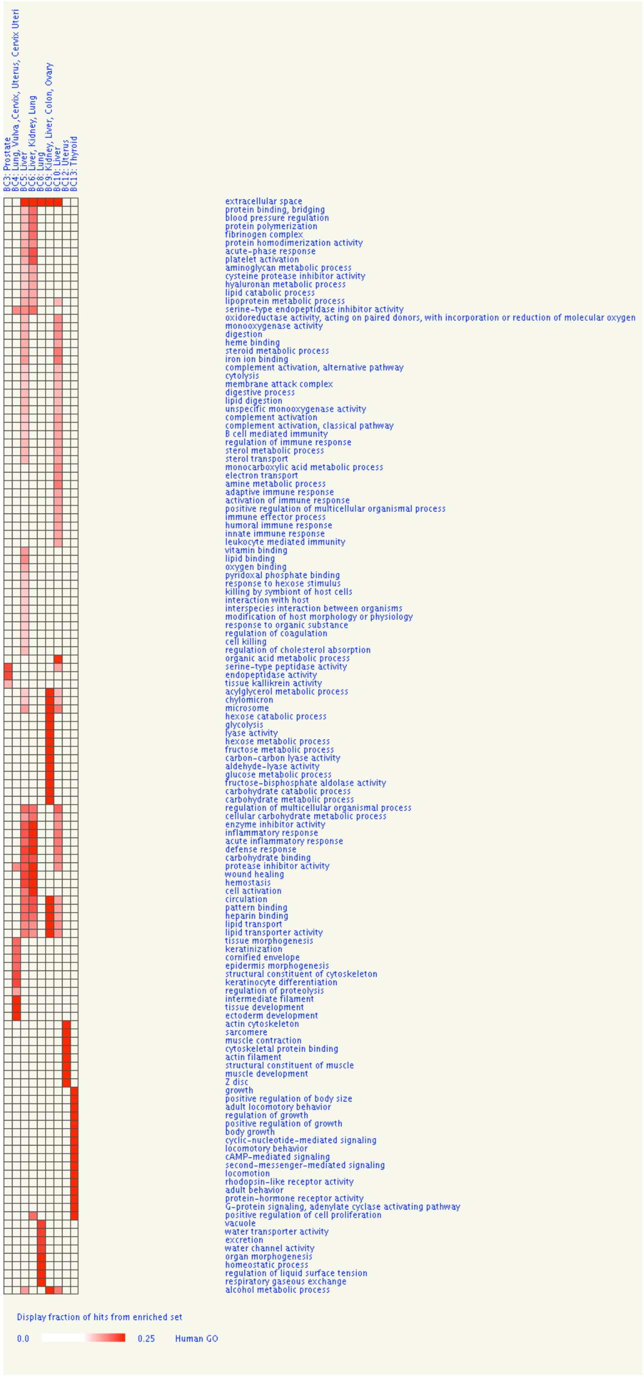

Figure 8: GO Enrichment of ExpO data
